# Minimally invasive anterior cervical foraminotomy for unilateral radiculopathy

**DOI:** 10.3171/2024.1.FOCVID23196

**Published:** 2024-04-01

**Authors:** Duncan J. Trimble, Dallas L. Sheinberg, Joseph A. Cochran

**Affiliations:** 1 Department of Neurosurgery, The University of Texas Health Science Center at Houston, Texas; and; 2 Memorial Hermann Southwest Hospital, Houston, Texas

**Keywords:** anterior cervical foraminotomy, ACF, minimally invasive, MIS, radiculopathy

## Abstract

Anterior cervical foraminotomy (ACF) is an alternative surgical option for the treatment of refractory unilateral radiculopathy due to disc herniation or spondylosis. The efficacy and adverse event rate in experienced practitioners are comparable to those of anterior cervical discectomy and fusion, total disc arthroplasty, and posterior foraminotomy. However, this technique has not been widely adopted, likely because of the proximity of the working zone and the vertebral artery. The authors present a detailed operative video of a patient successfully treated with an ACF. They also present a review of the ACF literature.

The video can be found here: https://stream.cadmore.media/r10.3171/2024.1.FOCVID23196

## Transcript

In this presentation, we will discuss minimally invasive C5–6 anterior foraminotomy with removal of a herniated disc fragment.

The patient is a 44-year-old gentleman with 6 months of worsening pain radiating from his neck to his right arm, right arm weakness, and numbness in the right thumb. His MRI demonstrates a right C5–6 foraminal disc herniation causing severe compression of the exiting C6 nerve. Our patient was amenable to surgery; however, he was highly motivated to return to work and to return to coaching and playing ice hockey as soon as possible.

### 0:52 Procedure Indications, Risks, Benefits, and Alternatives.

I decided to recommend a minimally invasive C5–6 anterior cervical foraminotomy, or ACF, with removal of herniated nucleus pulposus. Patients with symptomatic unilateral foraminal stenosis due to disc herniation and/or spondylosis are good candidates for this procedure.

Benefits of this procedure include direct neural decompression, sparing of the disc and motion segment, minimal postoperative restrictions with a quick return to work or return to play interval, and minimal cost due to absence of implanted hardware.^1^ Additionally, the anterior cervical approach is very familiar and well tolerated.

A risk of this procedure is that it is relatively unfamiliar; the aggregate published case series in the literature sum to less than 500 total cases.^2^ This unfamiliarity combined with the required bone removal near the vertebral artery intuitively carries a higher risk of vertebral artery injury than alternative procedures, which has likely limited the adoption of this technique. Additionally, the greater lateral retraction or the resection of a small portion of the longus colli carries a risk of a postoperative Horner’s syndrome.^1,2^ Last, given the disc-sparing nature of this technique, recurrent disc herniation is possible.^3^

The alternative procedures were not chosen for the same reasons discussed on the previous slide—we prioritized preserving the cervical disc and motion segment, avoiding hardware implantation, and minimizing tissue disruption, postoperative pain, and postoperative restrictions.

### 2:21 Positioning and Equipment.

For the procedure, the patient is positioned supine with the bed in slight Trendelenburg to orient the uncinate process in a vertical trajectory perpendicular to the floor. I use a 3-mm or 4-mm round diamond burr.

A three-blade MIS retractor is used; in this instance, the two medial blades that rest on the vertebral body are 50 mm and the lateral blade is 60 mm to allow effective retraction of the longus colli muscle. We have found the working corridor provided by a three-blade retractor to be sufficient, with the benefit of avoiding direct retraction on the esophagus and trachea, which we believe reduces postoperative dysphagia and hoarseness. To avoid pressure on the esophagus, the medial edges of the cephalad and caudal blades adjacent to the esophagus are elevated relative to the lateral edges of the blades, which are in direct contact with the spine. The use of tubular systems has been described and is a valid alternative.^4^

### 3:15 Preoperative Imaging.

Important anatomical structures for the procedure are highlighted here.

The longus colli muscle outlined in purple is mobilized to expose the C6 uncovertebral process. The lateral aspect of the C6 uncovertebral process outlined in orange is then drilled away using a coarse diamond drill. This opens a corridor medial to the vertebral artery that allows access to the neural foramen for removal of the herniated disc fragment and decompression of the nerve. If the operative level is at C6–7, the vertebral artery is frequently beneath the longus colli and above the C7 transverse process.^5^ As with any cervical spine procedure, the anatomy of the vertebral artery should be studied preoperatively to reduce the risk of injury, as variant anatomy is common.

### 4:01 Surgical Steps and Anatomy Review.

The key surgical steps are outlined here and will be better explained in the following slides and surgical video. These include an anterior cervical exposure with the skin incision on the symptomatic side, disconnection and mobilization of the longus colli laterally away from the spine, confirmation of the correct disc space, retractor insertion, and removal of the uncinate process with a high-speed drill to the posterior longitudinal ligament followed by palpation of the neural foramen and removal of the disc fragment.^1,4–6^

The slide demonstrates on AP intraoperative fluoroscopy and on a model where the small amount of bone is removed to access the disc fragment compressing the nerve. The fluoroscopy also demonstrates that the disc space remains essentially undisturbed.

This still shot from the intraoperative video demonstrates the position of the retractor blades. Note the longer retractor blade is moving the longus colli muscle out of the way, exposing the uncovertebral process. The C6 uncovertebral process is outlined in white, and the small amount of bone that will need to be removed is outlined in orange.

### 5:08 Operative Video Begins: Skin Incision to Retractor Placement.

We will now watch the operative video demonstrating this procedure.

We start with a 2.5-cm incision on the right side of the neck at the level of the C5–6 disc space. The skin and fat layer is sharply cut away from the platysma. That plane is developed with finger dissection. The platysma muscle is undermined and cut sharply in the direction of the muscle fibers. The avascular plane anterior to the sternocleidomastoid muscle and carotid artery is then developed until the longus colli muscle and disc are identified.

The longus colli muscle insertion on the vertebral body is then coagulated with bipolar cautery, and the longus colli muscle is cut sharply away from the spine. The muscle is further mobilized using a No. 1 Penfield instrument and a cervical marking pin is placed into the uncovertebral joint to confirm the correct level using fluoroscopy. I do not intentionally expose the vertebral artery, as was described in the original paper by Dr. Jho in 1996.^5^

The three-blade retractor is then placed with the longest blade, in this case a 60-mm blade used to retract the longus colli muscle away from the uncovertebral process.

### 6:29 Uncinate Process Removal and Discectomy.

The soft tissue overlying the uncovertebral joint is then removed using monopolar cautery. A 4-mm course diamond burr is used to remove the lateral aspect of the C6 uncovertebral process. We are staying lateral to the disc space. Copious amounts of irrigation are used to prevent thermal injury to the nerve. Venous bleeding can be encountered from the venous plexus surrounding the vertebral artery or from epidural veins in the neural foramen, but this is easily controlled with Floseal, or any other hemostatic agent. If for any reason vertebral artery injury is suspected, the use of Floseal is contraindicated. A small sharp angled curette is then used to probe the neural foramen and dissect the herniated disc fragment. A Kerrison punch is used to further widen the foraminal opening. A curet and nerve hook are used to mobilize the disc fragment before it is removed with a micro pituitary instrument. The neural foramen is then probed proximally and distally to make sure that there are no additional fragments.

### 8:01 Outcome.

The patient had immediate postoperative resolution of his radicular pain. He returned to work the following day and to playing ice hockey after 2 weeks. He remains symptom free at 6 months.

### 8:10 Literature Review: Technique.

The anterior cervical foraminotomy, also known as an anterior cervical microforaminotomy or ventral uncoforaminotomy, was first described by Dr. Jho in 1996.^5^ As previously mentioned, this technique has not been widely adopted, likely due to the proximity of the vertebral artery to the working zone. Saringer et al. published a modified technique in 2002 to limit the risk of vertebral artery injury.^6^ Modifications included not intentionally exposing the vertebral artery in the intertransverse space and leaving a thin wall of the lateral uncinate process intact between the working zone and the vertebral artery. Options for managing vertebral artery injury include direct open repair, endovascular stenting, and open or endovascular vessel sacrifice. Dr. Jho further refined his technique by adjusting the starting point of bony removal based on the level of pathology.^1^ Performing an ACF through a tube was first described in 2019 by Maduri et al.^4^

### 9:03 Literature Review: Outcomes.

This table of the published case series reveals overall good clinical results, low adverse event rates, and low rates of reoperation at the index level or at the adjacent level.^1–3,6–11^ No cases of vertebral artery injury were reported. Four cases of transient Horner’s syndrome were reported; one author modified his longus colli dissection technique from monopolar cautery to manual blunt dissection, a change that resulted in no further cases of Horner syndrome in that series.^2^ This is similar to the technique we employ. The rates of reoperation at the index level ranged from 1.3% to 9.5%, with an average of 3.6%. This is similar or slightly lower to the published rates of index-level reoperation for posterior cervical foraminotomy and slightly higher than the rate for ACDF.^12^

One case series was a significant outlier and demonstrated poor clinical results and a high index-level reoperation rate (26%), which was excluded from the above reoperation rate analysis. These authors abandoned the procedure and attributed their clinical results to a steep learning curve, which serves as a valid warning for the potential risks of implementing an unfamiliar procedure into practice.

## Disclosures

The authors report no conflict of interest concerning the materials or methods used in this study or the findings specified in this publication.

## Author Contributions

Primary surgeon: Cochran. Assistant surgeon: Trimble. Editing and drafting the video and abstract: Trimble, Sheinberg. Critically revising the work: Trimble, Sheinberg. Reviewed submitted version of the work: Trimble, Sheinberg. Approved the final version of the work on behalf of all authors: Trimble.

## Supplemental Information

### Patient Informed Consent

The necessary patient informed consent was obtained in this study.
